# Two Novel *ACTC1* Variants Cause Arthrogryposis Multiplex Congenita

**DOI:** 10.1111/cge.70218

**Published:** 2026-07-15

**Authors:** Lauren Kerr, Pierre Moffatt, Lauren Hyer, Sarah Nossov, Noémi Dahan‐Oliel, Frank Rauch

**Affiliations:** ^1^ Shriners Hospital for Children Montreal Canada; ^2^ Department of Human Genetics McGill University Montreal Canada; ^3^ Faculty of Dental Medicine and Oral Health Sciences McGill University Montreal Canada; ^4^ Shriners Hospital for Children Greenville South Carolina USA; ^5^ Shriners Hospital for Children Philadelphia Pennsylvania USA; ^6^ School of Physical and Occupational Therapy, McGill University Montreal Canada

**Keywords:** arthrogryposis multiplex congenita, cardiac alpha‐actin, immunofluorescence, western blot

## Abstract

Arthrogryposis multiplex congenita (AMC) is a group of conditions with congenital joint contractures in at least two body regions. *ACTC1* variants cause heart disorders but have also been reported in six families with AMC. We report on two individuals with AMC who were heterozygous for *ACTC1* missense variants (NM_005159.5; c.325G>A, p.Glu109Lys and c.650A>C, p.Lys217Thr). Individual 1 had scoliosis and no reported cardiac phenotype. Individual 2 had Shone's complex with coarctation of the aorta, which has not been reported in *ACTC1*‐related AMC before. In vitro testing demonstrated that p.Glu109Lys caused impaired actin filament assembly and reduced ACTC1 protein levels, effects not apparent for p.Lys217Thr. Our findings characterize two novel variants that cause *ACTC1*‐related AMC and expand the list of phenotypes observed in this disorder.

## Graphical abstract

We report on two individuals with arthrogryposis multiplex congenita who were heterozygous for *ACTC1* missense variants (NM_005159.5; c.325G>A, p.Glu109Lys and c.650A>C, p.Lys217Thr) and provide a characterization of these variants through in vitro studies. p.Glu109Lys caused impaired actin filament assembly and reduced ACTC1 protein levels, while p.Lys217Thr did not.

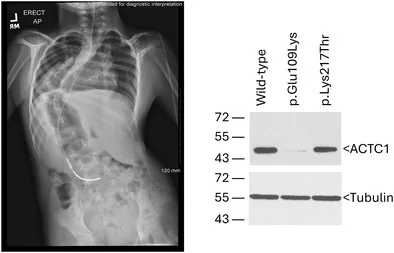

## 1 Introduction

*ACTC1*, the gene for cardiac alpha‐actin, has established gene‐phenotype relationships for the autosomal dominant disorders atrial septal defect type 5 (OMIM 612794), dilated cardiomyopathy type 1R (OMIM 613424), hypertrophic cardiomyopathy type 11 (OMIM 612098), and left ventricular noncompaction type 4 (OMIM 613424). A Noonan‐like phenotype can also occur [1]. Additionally, heterozygous missense variants in *ACTC1* have been reported in five families with arthrogryposis multiplex congenita (AMC) [2], a group of disorders with congenital joint contractures in at least two body regions [3]. Heart abnormalities were found in all individuals with echocardiography data. A case series on fetuses with AMC included one additional individual with a heterozygous *ACTC1* variant [4].

The amino acid sequence of ACTC1 is 98.9% homologous to that of ACTA1 (skeletal muscle alpha‐1 actin) [5]. *ACTA1* variants cause congenital myopathy types 2A, 2B, and 2C (OMIM 161800, 620 265, 620 278). Like *ACTC1*, *ACTA1* can also be implicated in congenital contractures. In a review on the molecular diagnosis of AMC, we found that *ACTA1* variants were detected in 10/991 families (1%) reported in cohort studies, making them a relatively common etiology (manuscript under review). To our knowledge, no data on the in vitro effects of AMC‐causing *ACTC1* variants have been published. In this report, we describe two families with heterozygous *ACTC1* missense variants causing AMC and present in vitro functional studies.

## 2 Patients and Methods

### 2.1 Subjects, Ethical Considerations, and Clinical Assessments

Both individuals were participants of the Shriners Hospitals for Children AMC RE^2^GISTRY [6]. IRB approval and informed consent were obtained as described in the Ethical Statement. Clinical information was collected and outcome measure surveys were administered as described [6].

### 2.2 DNA Sequencing

Genomic DNA was extracted from saliva and used for paired‐end genome sequencing (2 × 150 bp) with the NovaSeq 6000 Sequencing System (Illumina) at the Shriners Genomic Institute (Tampa, FL).

### 2.3 Protein Modeling

Protein modeling was done with ChimeraX software, version 1.11.1 [7]. Structure 93BR [8], chain A from Protein Data Bank (https://www.rcsb.org/) [9] was used.

### 2.4 In Vitro Studies

A pcDNA3.1‐based expression plasmid for human *ACTC1* (NM_005159.5) (OHu19196, GenScript, Piscataway, USA) was modified by PCR, deleting the C‐terminal FLAG tag and inserting it at the N‐terminus. *ACTC1* variants were introduced by site‐directed mutagenesis using PCR. Plasmid sequences were verified by Sanger sequencing. MC3T3‐E1 preosteoblastic cells were chosen for immunofluorescence due to their elaborate actin filament network. Cells were transfected with plasmids carrying wild‐type *ACTC1*, p.Glu109Lys, or p.Lys217Thr and processed for immunofluorescence. Protein extracts were prepared from transfected cells and Western blotting was performed as described [10].

## 3 Results

### 3.1 Individual 1

The girl was born by C‐section after 34 weeks of gestation (birth weight 1.64 kg, *z*‐score −1.5) as the first child of unrelated parents. Bilateral clubfeet, cleft palate, neck webbing, and spine deformity were noted. Her mother and one maternal aunt had been born with bilateral clubfeet and later developed scoliosis. The mother also had a history of cleft palate, but no history of cardiac issues.

Individual 1 began to walk and speak at age 2 years. She underwent cleft palate surgery at 2 years old and clubfoot surgery at 3 years. When examined at age 5 years, severe short stature (height: 90.5 cm, *z*‐score −5.4; weight: 12.8 kg, *z*‐score −4.2) and severe scoliosis (120 degrees) were noted (Figure 1A). At age 6 years, she underwent halo traction and scoliosis surgery with insertion of bilateral growing rods. At 8 years old, she had mild camptodactyly of both hands and mild contractures of the neck, left hip, and both knees and feet. She was highly independent for self‐care tasks. She had no significant mobility or cognitive difficulties. She reported back pain. At age 11 years, she was prescribed albuterol for wheezing. Menarche occurred at age 12 years. Cardiac problems were not noted.

**FIGURE 1:**
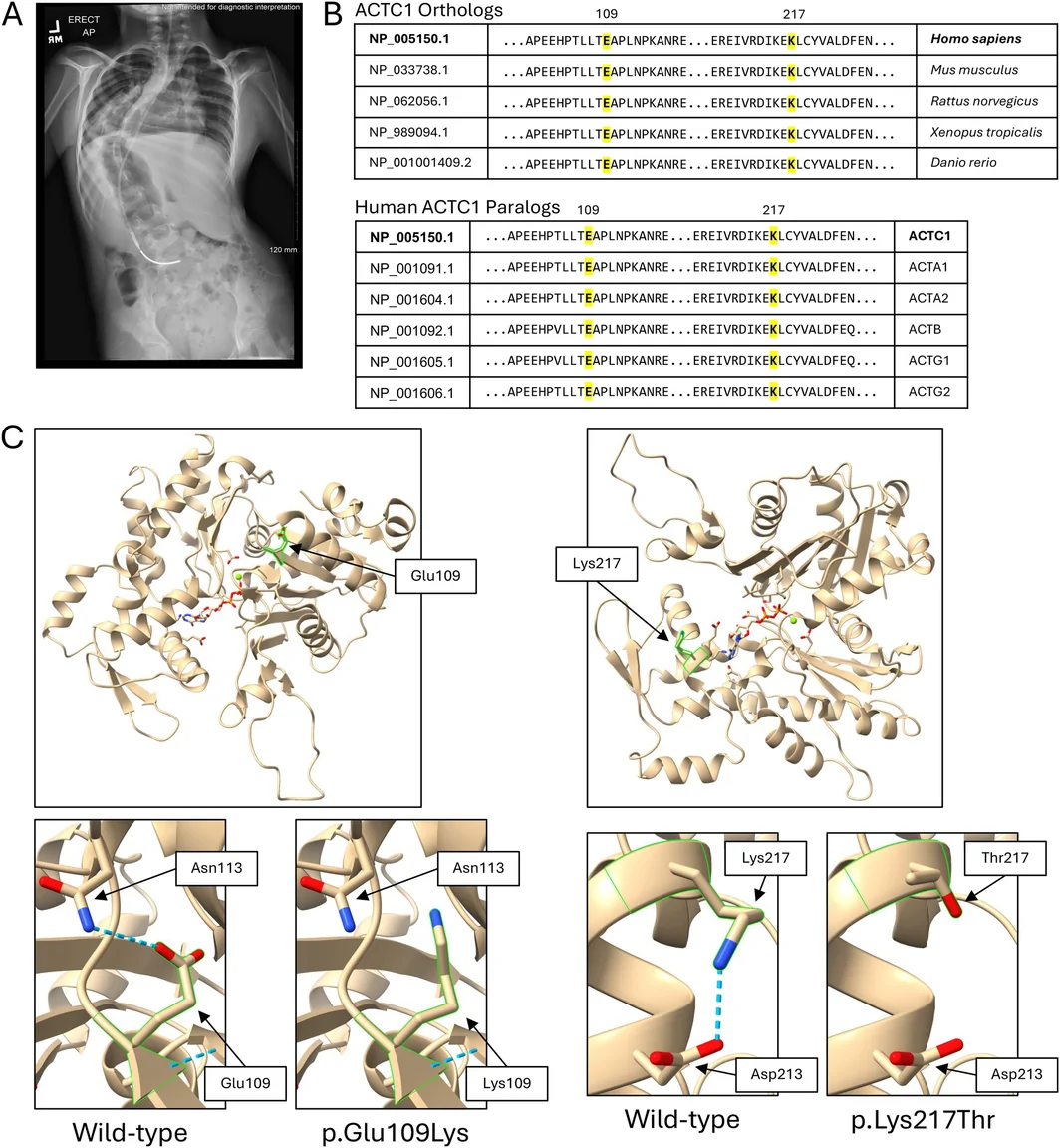
(A) Severe scoliosis in Individual 1. (B) Amino acid sequences of ACTC1 orthologs and human paralogs. Glu109 and Lys217 are highlighted. (C) Locations of Glu109 and Lys217 (top) and impact of p.Glu109Lys and p.Lys217Thr on amino acid interactions (bottom). The substitutions eliminate hydrogen bonds with the side chains of Asn113 and Asp213 (light blue dashed lines) and create hydrogen bonds with the backbones of Ala110 and Asp213, respectively (not shown). Structures in center: Red/orange/dark blue/beige = adenosine‐5′‐diphoshate; green = Mg^2+^.

### 3.2 Individual 2

The boy was born at 39 weeks of gestation (birth weight 3.94 kg, *z*‐score +1.4) as the first child of unrelated parents without family history of AMC. Limited range of motion in the wrists, fingers, thumbs, hips, knees, and bilateral clubfoot was noted. Echocardiography revealed Shone's complex, including coarctation of the aorta, which was repaired surgically.

During his first year of life, Individual 2 underwent bilateral plantar fascia release, bilateral posterior releases, and Achilles tendon lengthening with Ponseti casting. Examination at age 1 year showed clenched hands with deep single palmar creases, laterally deviated feet with overlapping toes and hypoplastic fifth toenails. Distal arthrogryposis was suspected. He had truncal hypotonia, mild lower extremity weakness and delayed achievement of gross motor milestones. At 2 years, he could stand with assistance. At age 3 years, height was 95.4 cm (*z*‐score −1.4) and weight was 14.2 kg (*z*‐score −1.1). Moderate contractures of wrists, thumb‐in‐palm deformities, and mild hip contractures were noted bilaterally. He crawled for mobility and required assistance with some self‐care tasks. He had no cognitive difficulties. At age 4 years, he began to walk independently. At age 5 years, he had further heart surgery and pacemaker insertion. At age 6 years, he underwent bilateral open tendon Achilles lengthening, anterior tibialis transfer, and right tibial rotational osteotomy. Spine x‐rays did not reveal scoliosis.

### 3.3 Genome Sequencing

Genome sequencing identified a heterozygous *ACTC1* variant (NM_005159.5; c.325G>A, p.Glu109Lys) in Individual 1 and her mother. The variant was absent from gnomAD v2.1.1, had an AlphaMissense score of 0.99 and a REVEL score of 0.827. It was classified as likely pathogenic with ACMG criteria (PM1, PM2, PP1, PP2, PP3). A de novo heterozygous *ACTC1* variant (NM_005159.5; c.650A>C, p.Lys217Thr) was found in Individual 2. It was absent from gnomAD and had an AlphaMissense score of 0.91 and a REVEL score of 0.918. It was also classified as likely pathogenic (PS2, PM1, PM2, PP2, PP3).

Both affected amino acids are conserved across ACTC1 orthologs and human ACTC1 paralogs (Figure 1B). For p.Glu109Lys, a likely pathogenic substitution has been reported in ClinVar at the homologous site in *ACTA1* (NM_001100.4; c.327G>C, p.Glu109Asp, Variation ID 2582813). Both ACTC1 variants alter hydrogen bonding (Figure 1C).

### 3.4 Immunofluorescence and Western Blotting

MC3T3‐E1 cells transfected with wild‐type ACTC1 displayed staining across the cytoplasm (Figure 2A). The overlap with phalloidin staining demonstrated that these bands corresponded to the actin filaments.

**FIGURE 2:**
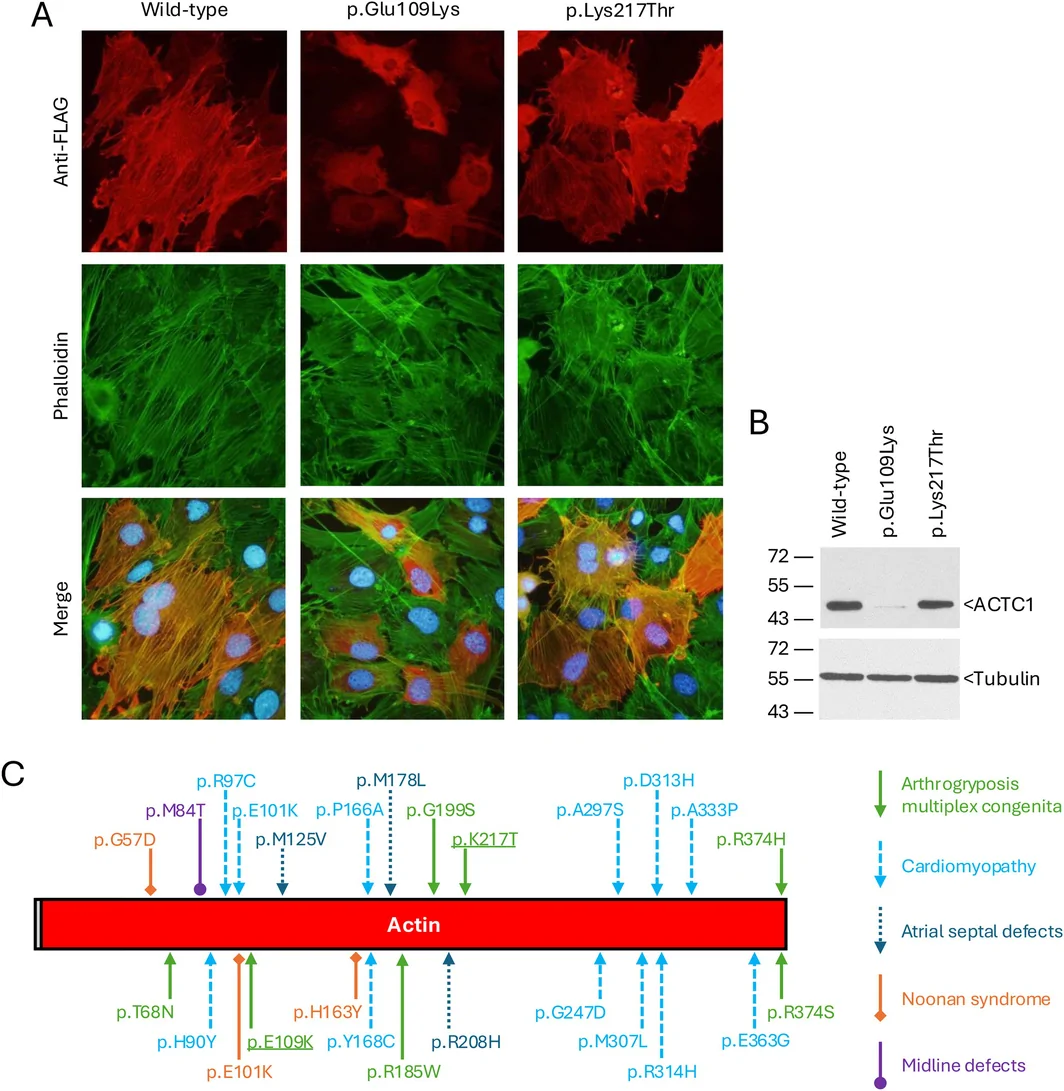
(A) Anti‐FLAG staining (red) of MC3T3 cells expressing wild‐type ACTC1, p.Glu109Lys, and p.Lys217Thr, with phalloidin staining targeting Actin filaments (green) and nuclear staining (blue) (40× magnification). (B) Western blot showing levels of the ACTC1 protein in cells with wild‐type ACTC1 and the two variants (top). Tubulin was used as a positive control (bottom). (C) ACTC1 variants reported in various phenotypes [1], with positions shown. Those contributed by this report are underlined.

Cells with ACTC1‐p.Glu109Lys had diffuse anti‐FLAG staining that overlapped poorly with phalloidin staining, suggesting impaired filament formation (Figure 2A). Western blotting demonstrated a reduced level of ACTC1‐p.Glu109Lys protein compared to wild‐type (Figure 2B), which may also affect filament formation.

For cells with ACTC1‐p.Lys217Thr, staining and protein levels appeared similar to those of cells with wild‐type ACTC1 (Figure 2A,B). Thus, p.Lys217Thr did not have clear effects on filament formation or protein availability.

## 4 Discussion

We report two novel *ACTC1* variants observed in unrelated individuals with AMC. p.Glu109Lys impacted ACTC1 protein levels and filament assembly in vitro, whereas p.Lys217Thr did not. Our findings contribute to the list of *ACTC1* missense variants linked to AMC (Figure 2C) and to the range of phenotypes observed (Table 1).

**TABLE 1:** Phenotypes reported in individuals with *ACTC1*‐related AMC.

Family	Chong et al. [2]					Turgut et al. [4]	This report	
	1	2	3	4	5	6	7	8
N presented	2	2	2	1	1	1	1	1
*ACTC1* variant NM_005159.5	c.595G>A, p.Gly199Ser	c.1120C>A, p.Arg374Ser	c.203C>A, p.Thr68Asn	c.553C>T, p.Arg185Trp	c.1121G>A, p.Arg374His	c.1121G>A, p.Arg374His	c.325G>A, p.Glu109Lys	c.650A>C, p.Lys217Thr
Joints involved	Elbows (1/2), fingers, hips, knees (1/2), ankle/foot (1/2)	Wrists, fingers, hips, knees, bilateral ankles/feet, toes	Elbows (1/2), fingers, ankle/foot (1/2), toes (1/2)	Elbows, hips, knees, ankle/foot	Elbows, wrists, fingers, hips, knees, ankle/foot, toes	Shoulders, elbows, wrists, knees, hands, ankles	Neck, fingers, one hip, knees, bilateral ankles/feet	Wrists, fingers, thumbs, hips, bilateral ankles/feet, toes
Heart involvement	ASD	−	ASD	ASD, VSD, dilated cardiomyopathy	Ductus venosus agenesis	−	−	Shone's complex, coarctation of aorta
Short stature	+ (ZNP)	+ (ZNP)	+ (1/2, ZNP)	+ (ZNP)	−	−	+ (*z*‐score −5.35)	−
Scoliosis	+	+	−	+	−	−	+	−
Other features	Neck pterygia	Neck pterygia	Neck pterygia, elbow pterygia (1/2)	Elbow pterygia, cleft palate	Neck pterygia	Multiple pterygia	Multiple pterygia, kyphosis, cleft palate, birth weight 1.64 kg (*z*‐score −1.5)	Birth weight 3.94 kg (*z*‐score + 1.4)

*Note:* + = presence; − = absence; ASD = atrial septal defect; VSD = ventricular septal defect; ZNP = *z*‐score(s) not provided. When a fraction is not specified, all individuals had that phenotype.

The individuals we describe had phenotypes resembling multiple pterygium syndrome and distal arthrogryposis, consistent with previous findings. Although heart defects and cardiomyopathy have been reported [2], Shone's complex, observed in Individual 2, has not been found in other individuals with *ACTC1*‐related AMC. In contrast, neither Individual 1 nor her mother had a history of cardiac problems. Heterogeneous cardiac phenotypes have been reported in 7/11 individuals thus far and may be absent or undetected in the remainder. Other characteristics, such as short stature and scoliosis, also appear inconsistently.

ACTC1‐p.Glu109Lys caused decreased ACTC1 protein levels and an apparent lack of organized actin filaments, while ACTC1‐p.Lys217Thr had expression and localization comparable to wild‐type ACTC1. Our observations on p.Glu109Lys support in silico findings that other *ACTC1* variants may affect thin filament assembly [2]. p.Glu109Lys could impede actin filament formation through structural instability and/or decreased protein availability for filament assembly, effects that support its pathogenicity.

p.Lys217Thr did not have a clear impact in our experimental system, but is likely to affect the function of ACTC1, considering that most residues in the actin proteins are completely conserved [5]. Indeed, known pathogenic missense variants in ACTC1 have been shown to assemble into filamentous actin while impacting other functions [11, 12]. Although the mechanism whereby p.Lys217Thr exerts its effect is not clear, the variant was de novo in the affected child and met ACMG criteria for being classified as likely pathogenic.

In conclusion, this report discusses two unrelated individuals with *ACTC1*‐related AMC, one of whom had Shone's complex and one of whom had no known heart defects. In vitro studies demonstrate that the *ACTC1* p.Glu109Lys variant affects actin filament assembly, while the p.Lys217Thr variant does not appear to do so.

## Author Contributions

**Lauren Kerr:** manuscript drafting, protein modeling. **Pierre Moffatt:** in vitro experiments, review, and editing. **Lauren Hyer:** clinical data verification, review, and editing. **Sarah Nossov:** clinical data verification, review, and editing. **Noémi Dahan‐Oliel:** study oversight, review, and editing. **Frank Rauch:** conceptualization, molecular diagnosis, writing, and editing.

## Funding

The study was funded by the Shriners Hospitals for Children (79137). L.K. is the recipient of a Canada Graduate Scholarship‐Master's award (Canadian Institutes of Health Research). N.D‐O is the recipient of a clinical research scholar award from the Fonds de recherche du Québec‐Santé.

## Ethics Statement

This study was approved by the McGill University Institutional Review Board (approval number: A08‐M30‐19B) on December 28, 2023. For US sites, approval was obtained from the Western Institutional Review Board (approval number: 20191755). The parents of both individuals in this report provided written informed consent for study participation and publication of clinical data.

## Conflicts of Interest

Frank Rauch: Angitia, BioMarin, Kirin, Ultragenyx: consulting fees. The other authors declare no conflicts of interest.

## Data Availability

Data sharing not applicable to this article as no datasets were generated or analysed during the current study.
